# Effect of Nonpharmaceutical Interventions in Preventing COVID-19 on the Circulation of Avian Influenza Virus in Wuhan, Hubei Province, China

**DOI:** 10.1155/2024/5528986

**Published:** 2024-05-25

**Authors:** Yixuan Wu, Wenhua Kong, Yijie Zhang, Sha Lu, Manqing Liu

**Affiliations:** ^1^ Department of Pathogen and Immunology Wuhan Center for Disease Control and Prevention Wuhan 430021 HubeiChina; ^2^ Infectious Disease Prevention and Control Institute Wuhan Center for Disease Control and Prevention Wuhan 430021 HubeiChina

## Abstract

**Background:**

In late 2019, several medical institutions in Wuhan, Hubei Province, China, reported cases of unexplained pneumonia. A novel coronavirus was isolated from human airway epithelial cells causing coronavirus disease-2019 (COVID-19). In recent years, many nonpharmaceutical interventions (NPIs) have been implemented to stop COVID-19 epidemic. This study aimed to explore the effect of NPIs on the circulation of avian influenza virus (AIV) in Wuhan.

**Materials and Methods:**

External environmental samples were collected and subjected to viral RNA extraction. Real-time quantitative polymerase chain reaction was used to detect the H5, H7, and H9 subtypes of AIV. Statistical analyses were performed using the chi-square test and binary logistic regression in SPSS 20.0 software.

**Results:**

A total of 2,451 external environmental samples were collected from seven districts from 2018 to 2022 in Wuhan, comprising 1,041 samples collected before COVID-19 and 1,410 samples after COVID-19. After COVID-19, the positive rate of AIV decreased significantly with the implementation of NPIs. The dominant subtype was the H9 subtype, followed by the H5 subtype. The positive rates of AIV in live poultry markets and poultry free-range sites were reduced significantly through the implementation of NPIs. Among the different sample types, higher positive rates of AIV were found in chopping boards, sewage, and cages. The positive rate of AIV was higher in trafficked source samples than that in autotrophic source samples.

**Conclusions:**

This study identified the characteristics of AIV in terms of different districts, surveillance sites, sample types, and bird sources in Wuhan. This study conducted a multifactorial analysis of the factors affecting AIV infection and provided a theoretical basis and guidance for the future prevention and control of AIV in Wuhan.

## 1. Introduction

Influenza A virus (IAV), a multihost virus, is classified into 18 and 11 subtypes based on its surface proteins, namely, hemagglutinin and neuraminidase, respectively [1]. Avian influenza virus (AIV) belongs to IAV and can be transmitted from wild birds to poultry and occasionally to humans, causing zoonotic infections [2]. In addition, AIV can be classified into low-pathogenicity AIV and high-pathogenicity AIV. Only the H5 and H7 subtypes of AIV are considered highly pathogenic [3, 4]. Although the H9 subtype is low pathogenicity, the expansion of its host species poses a potential threat to global health [5]. In 2009, the Chinese government established an agency responsible for monitoring AIV in poultry, wild birds, and humans. The agency collected samples every month in each province and reported them uniformly [6]. People mainly detect three subtypes of H5, H7, and H9 of AIV. Subtypes H5, H7, and H9 were first detected in China in 1996, 2013, and 1994, respectively [7, 8, 9]. The H5 and H9 subtypes are currently prevalent in China, and along with the epidemic of these two subtypes, other subtypes may also cause pandemics, such as H7N9, which was detected in Shanghai and Anhui, China, in 2013, and eventually caused several deaths [10, 11, 12]. The infection of H7N9 in humans has now been controlled in central urban areas of China [13]. In 2016, Wuhan Municipal People's Government issued a document called “Wuhan Livestock and Poultry Prohibited, Restricted and Suitable Breeding Areas Delineation and Implementation Program.” This document pointed out the scope and requirements of the “three zones.” The three zones were the prohibited, restricted, and suitable farming zones, respectively. The areas within the fourth-ring road of Wuhan were all prohibited breeding areas. In September 2017, poultry received bivalent H5/H7 inactivated vaccines in China [14]. According to the Wuhan Statistical Yearbook, the numbers of slaughtered poultries (10,000 heads) were 3,820.34, 4,088.50, 3,446.30, 3,675.68, and 3,737.47 in Wuhan from 2018 to 2022, respectively. The poultry trade in China was rarely reported, and the few regional reports were local surveys [15].

In late December 2019, several medical institutions in Wuhan, Hubei Province, China, reported cases of unexplained pneumonia [16]. Through epidemiological investigation, it was proposed that the cases might be related to a seafood market in Wuhan [17]. A novel coronavirus was isolated from human airway epithelial cells that caused coronavirus disease-2019 (COVID-19) [18]. COVID-19 and highly pathogenic AIV, such as H7N9, have similar characteristics, such as the possibility of developing severe respiratory diseases [19]. The Wuhan government attached great importance to COVID-19. The seafood market was disinfected to interrupt transmission. According to Circular of Wuhan City Command for Prevention and Control of Pneumonia Epidemic with COVID-19 (No.1), Wuhan's urban transportation was suspended, and airports and train stations were closed for leaving Wuhan on January 23, 2020, and controls on departures from Wuhan had been discontinued on April 8, 2020 (Circular of Hubei Provincial Command for Prevention and Control of Pneumonia Epidemic with COVID-19). Many nonpharmaceutical interventions (NPIs) were implemented to stop the COVID-19 epidemic, including mass quarantine, usage of face masks, and disinfection of the environment [20]. The NPIs mainly include personal prevention and group prevention. Personal precautions include wearing face masks, washing hands, maintaining appropriate social distances, and reducing participation in outdoor activities. Group precautions include home quarantine, closure of educational facilities, and prohibition of public gathering [21]. One study constructed the ordinary differential equation models based on four key NPIs in Wuhan at the time. The four key NPIs were the closure of the seafood market, the declaration of human-to-human transmission, the suspension of urban transportation, and the provision of additional medical resources. This study showed that cutting off environment-to-human and increasing isolation of infected people were effective in controlling transmission [22]. In addition, another study, which developed a modeling framework using epidemiological data and human movement data, found that there were 114,325 cases of COVID-19 in mainland China as of February 29, 2020, and there could be a 67-fold increase in the number of cases of COVID-19 if the country did not adopt the NPIs [23].

Wuhan is a megacity in Central China, with a resident population of nearly 14 million. Wuhan, as the largest water, land, and air transportation hub in the inland region, is the shipping center of the middle reaches of the Yangtze River, which, as a special geographical location and large population base, makes it an important surveillance site for AIV. We compared the positive rates of AIV in various regions of Wuhan before and after COVID-19 and explored the effects of NPIs on the changes in AIV. Based on these data, we recommend that government should strengthen the monitoring of AIV in various districts in Wuhan.

## 2. Results

### 2.1. Detection of AIV in Environmental Samples from 2018 to 2022

From 2018 to 2022, we collected 2,451 external environmental samples for the detection of AIV, including H5, H7, and H9 subtypes. The non-H5/7/9 subtypes were referred to here as Untyped. Due to the epidemic of COVID-19, we did not collect external environmental samples from February to May 2020. Based on the outbreak of COVID-19, we divided the results into two stages, namely, before COVID-19 (January 2018–January 2020) and after COVID-19 (June 2020–December 2022). We collected 1,041 and 1,410 samples for the before and after COVID-19 stages, respectively. After COVID-19, a large number of NPIs were implemented. The positive rate of AIV (16.03%) decreased significantly with NPIs (*χ*^2^ = 62.959, *p*  < 0.001). The positive rate of AIV in 2020 (11.11%) declined dramatically compared with that in 2019 (32.81%) and slightly increased in the following 2 years. However, the positive rate of AIV was much lower in 2022 (18.33%) than that in 2019 (32.81%). The implementation of NPIs reduced the positive rate of H5 and H9 coexistence (1.77%; *χ*^2^ = 17.606, *p*  < 0.001). However, the main subtypes of AIV were still H9, followed by H5. Only one coexistence of H7 and H9 was detected in 5 years, which was not included in the statistical results.  Table 1 showed the yearly positive rates of AIV, H5, H9, Untyped, and the coexistence of H5 and H9 in environment samples.

### 2.2. Positive Rates of AIV in Different Districts of Wuhan

**Figure 1:**
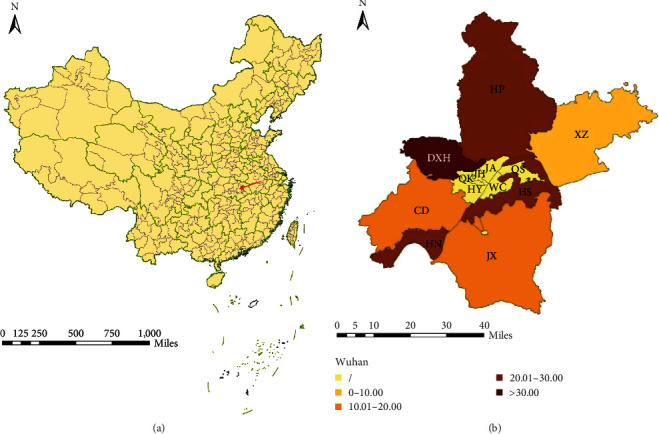
Geographical distributions of AIV with different positive rates (%). (a) The area indicated by the red arrow is Wuhan. (b) The different colors represent the corresponding range of the positive rates of AIV. The darker color means higher positive rates. A total of six districts of Wuhan were not monitored for AIV and were replaced by a slash. Abbreviations: CD, Caidian; DXH, Dongxihu; HN, Hannan; HP, Huangpi; HS, Hongshan; HY, Hanyang; JA, Jiang'an; JH, Jianghan; JX, Jiangxia; QK, Qiaokou; QS, Qingshan; WC, Wuchang; and XZ, Xinzhou. The maps were selected from the website, called “datav.geoatlas”.

Wuhan is located in Central China (Figure 1(a)). Seven districts were selected for monitoring, namely Hongshan, Caidian, Dongxihu, Huangpi, Jiangxia, Xinzhou, and Hannan districts. The AIV was detected in Dongxihu District (42.45%) with the highest positive rate, while Xinzhou District (8.75%) had the lowest positive rate of AIV. The coexistence of H5 and H9 was mostly found in Hannan District (7.60%) (Table 2). The positive rates of AIV decreased significantly in all districts after the implementation of NPIs. The geographical distribution of the positive rates of AIV is shown in Figure 1(b).

### 2.3. Positive Rates of AIV in Different Monitoring Sites

**Figure 2:**
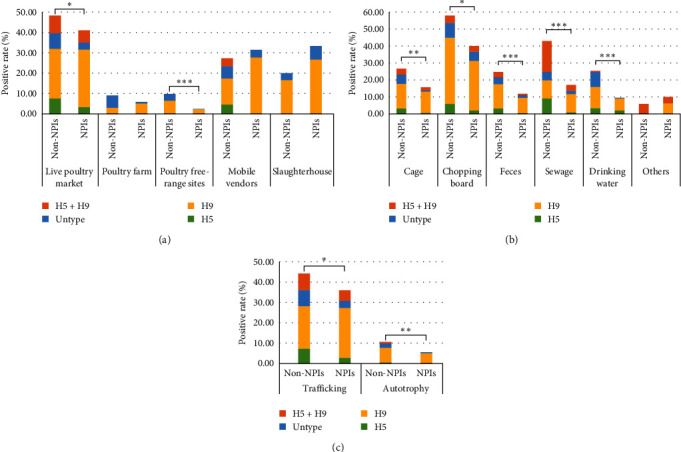
Differences in AIV positive rates (%) by implementing NPIs. (a) The *X*-axis represents different monitoring sites, and the *Y*-axis represents positive rates (%). (b) The *X*-axis represents different sample types, and the *Y*-axis represents positive rates (%). (c) The *X*-axis represents the origins of different birds, and the *Y*-axis represents positive rates (%). The red bar represents coexistence of H5 and H9, the blue bar represents Untyped samples, the yellow bar represents H9 subtype, and the green bar represents H5 subtype. The non-NPIs correspond to the stage before COVID-19, and the NPIs correspond to the stage after COVID-19. The period from January 2018 to January 2020 was defined as before COVID-19, while the period from June 2020 to December 2022 was defined as after COVID-19. The non-H5/7/9 subtypes were referred to as Untyped. The chi-square test was used to analyze the significant difference. Statistical significance was considered at *P*  < 0.05. Asterisks represent statistically significant differences between different groups:  ^*∗*^*P*  < 0.05,  ^*∗∗*^*P*  < 0.01, and  ^*∗∗∗*^*P*  < 0.001.

This study included six surveillance sites, namely, live poultry markets, poultry farms, free-range poultry sites, mobile vendors, slaughterhouses, and wild bird habitats. A total of 10 samples with unknown surveillance site information were excluded from Table 3. Live poultry markets (44.89%), mobile vendors (28.49%), and slaughterhouses (28.00%) were the surveillance sites with high positive rates for AIV. The AIV had not been detected in wild bird habitats. Table S1 shows the detection of different subtypes of AIV at different monitoring sites. The H5 subtype occurred only in two sites, which were live poultry markets (5.61%) and mobile vendors (3.23%). The H9 subtype occurred mainly in live poultry markets (26.26%), slaughterhouses (22.67%), and mobile vendors (17.20%). The coexistence of H5 and H9 occurred mainly in live poultry markets (7.41%) and mobile vendors (2.69%). With NPIs, the positive rates of AIV declined in live poultry markets, poultry farm, and poultry free-range sites, and grew in mobile vendors and slaughterhouse. However, the changes in the rates of AIV positivity were statistically different only in live poultry markets (*χ*^2^ = 4.785, *p*=0.029) and poultry free-range sites (*χ*^2^ = 21.332, *p* < 0.001). The positive rates of AIV dropped the most in poultry free-range sites, by 7.38%, followed by 7.30% in live poultry markets (Figure 2 (a)).

### 2.4. Positive Rates of AIV in Different Sample Types

Samples were collected from different sources, including cages, chopping boards, feces, sewage, and drinking water. The high positive rates of AIV were detected in samples collected from chopping boards (45.66%), sewage (26.69%), and cages (21.04%) (Table 3). Table S2 shows the detection of different subtypes of AIV in different samples. The H5 subtype was detected in sewage samples with the highest positive rate (3.99%). The H9 subtype was detected in chopping board samples with the highest positive rate (32.42%). The highest positive rate of H5 and H9 coexistence was found in sewage samples (8.90%). The positive rates of AIV decreased in all samples except for other types. The implementation of NPIs reduced the positive rates of AIV significantly in cages (*χ*^2^ = 11.273, *p*=0.001 < 0.01), chopping boards (*χ*^2^ = 6.152,*p*=0.013 < 0.05), feces (*χ*^2^ = 20.757, *p* < 0.001), sewage (*χ*^2^ = 26.092, *p* < 0.001), and drinking water (*χ*^2^ = 19.057, *p* < 0.001). The positive rates of AIV declined the most in sewage samples, by 25.91%, followed by 17.97% in chopping board samples. The lowest decrease of AIV positivity was 11.24% in cage samples (Figure 2(b)).

### 2.5. Positive Rates of AIV in Birds of Different Origins

According to the origins of birds, samples were classified as trafficking, autotrophy, and wild. One hundred samples with unknown bird origin information were excluded from Table 3. The AIV was not detected in wild source samples. The positive rate of AIV was much higher in trafficked source samples (40.20%) than that in autotrophic source samples (7.24%; *χ*^2^ = 332.724, *p* < 0.001). Table S3 shows the detection of different subtypes of AIV in birds of different origins. The positive rates of various subtypes of AIV were all higher in trafficked source samples than that in autotrophic source samples. The implementation of NPIs reduced the positive rates of AIV significantly in trafficked source samples (*χ*^2^ = 6.652, *p*=0.010 < 0.05) and autotrophic source samples (*χ*^2^ = 9.720, *p*=0.002 < 0.01). The positive rates of AIV declined the most in trafficked source samples, by 8.08%, followed by 5.03% in autotrophic source samples (Figure 2(c)).

### 2.6. Multifactorial Analysis of Factors Affecting AIV Infection

The chi-square test was used to analyze the factors that might contribute to AIV and showed that different years (*χ*^2^ = 72.500, *p* < 0.001), monitoring sites (*χ*^2^ = 531.653, *p* < 0.001), sample types (*χ*^2^ = 101.356, *p* < 0.001), and sample origins (*χ*^2^ = 460.005, *p* < 0.001) were all statistically significant. Using all four of these factors as independent variables, the dependent variable was whether or not AIV was detected. The results of the analysis using binary logistic regression were displayed in Table 4. The probabilities of detecting AIV in 2020 (*p* < 0.001) and 2021 (*p* < 0.001) were 0.378 and 0.464 times higher than that in 2018, respectively. The probabilities of detecting AIV in the four monitoring sites, namely, poultry farm (*p* < 0.001), poultry free-range sites (*p* < 0.001), mobile vendors (*p*=0.020 < 0.05), and slaughterhouse (*p* < 0.001) were 0.137, 0.124, 0.625, and 0.314 times higher than that in live poultry markets, respectively. The AIV was 2.357 times more likely to be detected in the chopping board samples (*p* < 0.001) than that in the cage samples. The probabilities of detecting AIV in the autotrophy samples (*p* < 0.001) were 0.347 times higher than that in trafficking samples.

## 3. Discussion

According to the Wuhan Statistical Yearbook, the number of slaughtered poultry in Wuhan increased in 2019 compared to that in 2018, with a significant decline in 2020 (15.71% compared to 2019), followed by upward trends seen in the following 2 years. But the total number of slaughtered poultry in 2022 remained less than that in 2018. A national survey study showed that the positive rate of AIV was 22.57% from 2014 to 2018 in China, and the positive rate in Hubei Province, where Wuhan is located, was lower than 22.57% [6]. This study showed that the positive rates of AIV were 28.32% in 2018 and 32.81% in 2019. In 2020, after the COVID-19 outbreak, the positive rate of AIV in Wuhan dropped dramatically to 11.11%. The Chinese government identified COVID-19 as a Class B infectious disease but took preventive measures normally used for Class A infectious disease. After COVID-19, the government closed down the relevant seafood markets for the first time and thoroughly decontaminated them. All wild animal transactions were also banned [24]. The Wuhan government closed the city transportation and airports and train stations to minimize the movement of people and take environmental disinfection and sterilization measures in many markets or public places. The general public had been asked to take strict personal precautions, including wearing masks in public places, washing hands frequently, and maintaining social distancing. Studies have shown that these NPIs reduced the spread of some respiratory diseases, such as influenza virus [25]. As expected, the positive rate of AIV in Wuhan in 2020 was much lower than that in 2019. In April 2020, controls on departures from Wuhan have been discontinued. However, the Wuhan government still enforced NPIs strictly, and the residents were particularly aware of the importance of NPIs for their protection. The positive rates of AIV were still much lower in 2021 and 2022 than that in 2019. Nanchang, the capital of Jiangxi Province, China, had a higher positive rate of AIV after COVID-19 than before, which might be caused by the large accumulation of live poultry in farms [26].

In 2016, the government of Wuhan introduced a policy to ban live poultry farming in urban areas within the fourth-ring road. There are 13 administrative divisions in Wuhan, the remaining six districts are Jiang'an, Jianghan, Wuchang, Qiaokou, Hanyang, and Qingshan, respectively, all of which are central urban areas and within the fourth-ring road. The positive rate of AIV in Wuhan was unevenly distributed in different districts, with the highest positive rate in Dongxihu District and the lowest positive rate in Xinzhou District. During the 5 years of surveillance, the AIV was dominated by the H9 subtype in Wuhan. Because of its low pathogenicity, the H9 subtype is not under primary surveillance and control in many countries. A study showed that H9N2 had replaced H5N6 and H7N9 as the dominant AIV subtype in China and that almost all H9Ny subtypes were easier than other subtypes to bind to human-type receptors [27]. The prevalence of H9N2 increased in the serum of susceptible people [28]. The annual isolation rate of H9N2 increased in Southern China and antigenic drift appeared [29]. In addition, H5 subtype of AIV was also detected in this surveillance. The H5 subtype was detected alone or coexisted with the H9 subtype. Studies have shown that wild birds could transmit H5N6, a highly pathogenic AIV, to poultry along their flight paths. At the same time, the virus was also highly pathogenic to mice, suggesting that it posed a potential threat to mammals [30]. We had not detected the H7 subtype in this study. Only one case of coexistence with the H7 and H9 subtypes was detected in 5 years. Studies have shown that coexistence with H7N9 and H9N2 viruses may lead to the emergence of new recombinant viruses in chickens, and these new recombinants had the potential for further transmission [31]. Although the main prevalent AIV now is the H9 subtype in Wuhan, we cannot ignore the monitoring for highly pathogenic AIV. In 2013, the first human case of H7N9 infection was reported in China. There had been five outbreaks of H7N9 in China, affecting most cities [13]. The Chinese guidelines on diagnosis and treatment of infection with the H7N9 virus were introduced in 2017 [32]. Europe had at least 10 incursions of H5 subtype, resulting in massive poultry and wild birds deaths [33]. Highly pathogenic AIV posed threats to the public health and poultry industry, because of its rapid evolution, enhanced virulence, and efficient transmission [3].

The amount of live poultry trade were an important factor contributing to the spread of AIV, and poultry were mainly transported along domestic railroads or national highways [34]. So poultry spread AIV regionally, which meant the virus was more likely to originate and migrate from and to other places that belonged to the same community [35]. One study found that in Guangxi, China, different districts were highly connected through live poultry transportation, creating conditions for rapid spread of the AIV throughout the province [36]. In this study, we conducted surveillance at different sites, and the highest positive rate of AIV was detected in live poultry markets, followed by mobile vendors and slaughterhouses. The samples collected from live poultry markets showed the highest positive rates for the H5 and H9 subtypes and the coexistence of the two. Many epidemics of AIV were generated in live poultry markets, and the positive rate of AIV would reduce after the closure of live poultry markets while the risk of human infection with AIV would be also reduced significantly. In June 2013, the risk of human infection was reduced by 97%–99% after closing live poultry markets in four cities [37]. This showed that the measures taken in NPIs, such as disinfestation and even closure of live poultry markets, stopped trade and the spread of the AIV in live poultry. The closure of all live poultry markets in the city was a very effective measure to prevent the spread of AIV, and extending the closure period and considering permanent closure would contribute to the controlling of AIV circulation [38]. Once a confirmed case of people who infected AIV had been discovered, the government played a key role in the timely implementation of closing down live poultry markets [32]. One study found that workers in live poultry markets had low education, insufficient awareness of AIV, and inadequate preventive behaviors [39]. Therefore, strengthening the dissemination of professional knowledge and training on protective measures for such workers were important parts of preventing the spread of AIV. The live poultry markets in Wuhan are mainly in distant urban areas. After the outbreak of COVID-19, the government paid more attention to control live poultry markets. Extensive environmental disinfection and prohibition of irregularities both resulted in lower positive rates of AIV in live poultry markets after COVID-19 than before.

We collected different types of samples and found these collected from chopping boards had the highest positive rate of AIV, followed by sewage, which was consistent with the previous results of Hubei Province [40]. Influenza virus can infect various hosts and undergo genetic recombination, and it is transmitted primarily through air and water [41]. After COVID-19, many NPIs were implemented, and people's awareness of personal protection increased, thus resulting in much lower positive rates of AIV for almost all types of samples than before. We found that the positive rate of AIV in birds from autotrophy was much lower than that in birds from trafficking, because trafficked birds had different origins, species, and irregular transport conditions. In contrast, self-raised birds were more homogeneous and generally vaccinated. Poultry were often transferred by vehicle during the poultry trade, which increased the risk of AIV transmission significantly [42]. Studies have confirmed that the movement of live birds increased the risk of transmission of AIV, and the removal of infected birds could reduce this risk somewhat [43]. At the same time, the coexistence of H5 and H9 was more likely to occur in poultry samples from trafficking. After COVID-19, many poultry shipment routes were restricted and self-raised poultry sites were frequently disinfected. Therefore, the positive rates of AIV in both trafficking and self-raised sources of poultry samples were lower after COVID-19 than before.

There were several deficiencies in this study. First, the study lacked the introductions of the number of markets and poultry population. In the future, strengthening cooperation with relevant departments will be a good way to obtain more relevant information. Second, there were some samples of unknown subtypes in the results. We planned to conduct further research on these samples. These samples will be first cultured, followed by second-generation sequencing. Third, this monitoring was mainly focused on the distant urban areas of Wuhan, because the live poultry markets, poultry farms, and slaughterhouses were mainly located in the distant urban areas. However, monitoring of the central areas, e.g., mobile vendors, should be strengthened in the future. Fourth, water was likely to be an important route of the epidemic transmission of AIV [44]. Wuhan has more than 100 lakes. In this study, we collected 211 samples from several lakes over 5 years, and no AIV was detected. From November 2018 to March 2019, some scholars conducted surveillance for wild birds in lakes and wetlands in Central China and found that the positive rate of AIV was 1.38% [45]. In the future, we should strengthen the surveillance in wild lakes during the migration season.

## 4. Materials and Methods

### 4.1. External Environmental Sample Collection

This surveillance was conducted from January 2018 to December 2022, and samples were not collected from January 2020 to April 2020, because of the outbreak of COVID-19. In 2016, the government of Wuhan introduced a policy to ban live poultry farming in urban areas within the fourth-ring road. In the same year, Hubei Province introduced a monitoring program. The program required that at least 10 samples that distributed in different places and among different types were collected once a month, and not less than 120 samples throughout the year. The monitoring points were set up according to different monitoring sites. For example, each live poultry market, poultry farm, slaughterhouse, or wild bird habitat was one monitoring point. In areas that contained many poultry free-range sites, each natural village was set as a monitoring point. In the high-occurrence season of AIV (October of this year to March of the following year), at least 10 samples would be collected in each month of each distant urban area, and during the rest of the year, each month we selected one area to collect at least 10 samples. The collection of samples should be distributed among different monitoring sites, with 2–3 samples per site, avoiding sampling within 2 days of disinfection of the monitoring site. The external environmental samples were collected in 10 ml standard virus sampling tubes (noninactivated) or 15 ml centrifuge tubes for drinking water/sewage samples, and transferred to the laboratory within 2 days following the standardized transfer process.

### 4.2. RNA Extraction

The samples were first centrifuged at 3,000 rpm for 10 min, and subjected to viral RNA extraction with a viral DNA/RNA extraction kit (CDC) (CqEx-DNA/RNA virus, Tianlong Science and Technology Co., Ltd., Xi'an, China) using a fully automated nucleic acid extractor (Generotex 96, Tianlong Science and Technology Co., Ltd., Xi'an, China). The total RNA was stored at −70°C for detection.

### 4.3. Real-Time Quantitative PCR

A real-time quantitative polymerase chain reaction assay was used to detect H5, H7, and H9 subtypes of IAV. The reagent was purchased from Beijing Applied Biological Technologies Co., Ltd. (A2044, Beijing, China). Specific operations were performed under the manufacturer's instructions.

### 4.4. Map Plotting

We drew the map using ArcGIS version 10.2 (Environmental Systems Research Institute, Inc., California, USA). The maps were selected from the website, which called “datav.geoatlas.” The different colors represented the corresponding range of the positive rates of AIV.

### 4.5. Statistical Analysis

We used Excel 2016 software to enter and organize the data. We used IBM SPSS 20.0 software (IBM Corp., Armonk, NY, USA) for statistical analysis. Most of the data were expressed as mean ± SD. The chi-square test was used to analyze the significant differences. The binary logistic regression was used to analyze the effects of multifactorial. Statistical significance was considered at *P* < 0.05. Asterisks represent statistically significant differences between different groups:  ^*∗*^*P* < 0.05,  ^*∗∗*^*P* < 0.01, and  ^*∗∗∗*^*P* < 0.001.

## 5. Conclusions

In summary, this study found that the positive rate of AIV dropped significantly after 2020, which might be associated with the NPIs caused by the COVID-19 outbreak. Further study is necessary to explore the direct and detailed effect of NPIs on the positive rate of AIV, including virus mutation in the long term.

## Figures and Tables

**Table 1:** AIV, H5, H9, Untyped, and coexistence of H5 and H9 nucleic acid positive rates in different years and stages.

Categories	*N*	AIV (%)	H5 (%)	H9 (%)	Untyped (%)	H5 + H9 (%)
Years						
2018	671	190 (28.32)	23 (3.43)	91 (13.56)	51 (7.60)	25 (3.73)
2019	320	105 (32.81)	15 (4.69)	61 (19.06)	7 (2.19)	22 (6.88)
2020	270	30 (11.11)	4 (1.48)	24 (8.89)	0 (0)	2 (0.74)
2021	410	64 (15.61)	0 (0)	60 (14.63)	3 (0.73)	1 (0.24)
2022	780	143 (18.33)	14 (1.79)	87 (11.15)	18 (2.31)	24 (3.08)
Total	2,451	532 (21.71)	56 (2.28)	323 (13.18)	79 (3.22)	74 (3.02)
*χ*^2^	—	72.500	23.406	17.665	61.567	32.973
*p*	—	<0.001	<0.001	0.001	<0.001	<0.001
Periods						
Non-NPIs	1,041	306 (29.39)	42 (4.03)	157 (15.08)	58 (5.57)	49 (4.71)
NPIs	1,410	226 (16.03)	14 (0.99)	166 (11.77)	21 (1.49)	25 (1.77)
*χ*^2^	—	62.959	24.817	5.730	31.993	17.606
*p*	—	<0.001	<0.001	0.017	<0.001	<0.001

Non-NPIs corresponded to the stage before COVID-19, and NPIs corresponded to the stage after COVID-19. The period from January 2018 to January 2020 was defined as before COVID-19, while the period from June 2020 to December 2022 was defined as after COVID-19. The non-H5/7/9 subtypes were referred to as Untyped.

**Table 2:** AIV, H5, H9, Untyped, and coexistence of H5 and H9 nucleic acid positive rate in different districts of Wuhan (positive rates (%) = (number of positive samples/number of samples collected) × 100).

Categories	HS	CD	DXH	HP	JX	XZ	HN
Subtypes							
H5	1.93 (11/570)	1.99 (8/403)	4.72 (15/318)	3.33 (12/360)	1.62 (5/309)	0.62 (2/320)	1.75 (3/171)
H9	13.51 (77/570)	12.16 (49/403)	29.56 (94/318)	11.39 (41/360)	5.18 (16/309)	5.94 (19/320)	15.79 (27/171)
Untyped	4.04 (23/570)	2.23 (9/403)	5.35 (17/318)	5.00 (18/360)	2.27 (7/309)	0.31 (1/320)	2.34 (4/171)
H5 + H9	5.26 (30/570)	1.99 (8/403)	2.83 (9/318)	1.39 (5/360)	0.97 (3/309)	1.87 (6/320)	7.60 (13/171)
Total	24.74 (141/570)	18.36 (74/403)	42.45 (135/318)	21.11 (76/360)	10.03 (31/309)	8.75 (28/320)	27.49 (47/171)
Periods							
Non-NPIs	37.33 (56/150)	23.53 (36/153)	45.27 (67/148)	31.11 (56/180)	17.45 (26/149)	13.33 (20/150)	40.54 (45/111)
NPIs	20.24 (85/420)	15.20 (38/250)	40.00 (68/170)	11.11 (20/180)	3.12 (5/160)	4.71 (8/170)	3.33 (2/60)
* χ* ^2^	17.350	575.202	569.607	601.045	598.128	578.116	653.225
* p*	<0.001	<0.001	<0.001	<0.001	<0.001	<0.001	<0.001

Non-NPIs corresponded to the stage before COVID-19, and NPIs corresponded to the stage after COVID-19. The period from January 2018 to January 2020 was defined as before COVID-19, while the period from June 2020 to December 2022 was defined as after COVID-19. The non-H5/7/9 subtypes were referred to as Untyped. *Abbreviations*. CD, Caidian; DXH, Dongxihu; HN, Hannan; HP, Huangpi; HS, Hongshan; JX, Jiangxia; and XZ, Xinzhou.

**Table 3:** AIV nucleic acid positive rate of different monitoring sites, sample types, and birds origins (positive rates (%) = (number of positive samples/number of samples collected) × 100).

Categories	Non-NPIs	NPIs	Total	Mean ± SD	*χ* ^2^	*p*
Monitoring sites						
Live poultry markets	48.31 (229/474)	41.01 (171/417)	44.89 (400/891)	43.16 ± 9.34	4.785	0.029
Poultry farm	9.09 (9/99)	5.88 (8/136)	7.23 (17/235)	5.62 ± 6.06	0.879	0.348
Poultry free-range sites	9.77 (21/215)	2.39 (15/628)	4.27 (36/843)	3.36 ± 5.36	21.332	<0.001
Mobile vendors	27.27 (36/132)	31.48 (17/54)	28.49 (53/186)	27.57 ± 20.87	0.333	0.564
Slaughterhouse	20.00 (6/30)	33.33 (15/45)	28.00 (21/75)	19.67 ± 16.35	1.587	0.208
Wild bird habitat	0 (0/81)	0 (0/130)	0 (0/211)	/	/	/
Sample types						
Cage	26.95 (76/282)	15.71 (49/312)	21.04 (125/594)	21.37 ± 6.06	11.273	0.001
Chopping board	57.97 (40/69)	40.00 (60/150)	45.66 (100/219)	44.02 ± 17.63	6.152	0.013
Feces	24.53 (92/375)	12.11 (51/421)	17.96 (143/796)	17.11 ± 9.01	20.757	<0.001
Sewage	42.98 (52/121)	17.07 (35/205)	26.69 (87/326)	27.17 ± 17.49	26.092	<0.001
Drinking water	25.42 (45/177)	9.50 (23/242)	16.23 (68/419)	16.80 ± 12.04	19.057	<0.001
Others	5.88 (1/17)	10.00 (8/80)	9.28 (9/97)	8.67 ± 10.40	0.282	0.595
Birds origins						
Trafficking	44.22 (218/493)	36.14 (176/487)	40.20 (394/980)	38.36 ± 10.71	6.652	0.010
Autotrophy	10.59 (41/387)	5.56 (43/773)	7.24 (84/1,160)	7.90 ± 3.49	9.720	0.002
Wild	0 (0/81)	0 (0/130)	0 (0/211)	/	/	/

Non-NPIs corresponded to the stage before COVID-19, and NPIs corresponded to the stage after COVID-19. The period from January 2018 to January 2020 was defined as before COVID-19, while the period from June 2020 to December 2022 was defined as after COVID-19. The non-H5/7/9 subtypes were referred to as Untyped. SD, standard deviation.

**Table 4:** Multifactorial analysis of binary logistic regression for AIV nucleic acid positive rate.

Independent variables	*β*	SE	*p*	*OR*	95% CI for OR	
					Lower	Upper
Year						
2019	−0.011	0.173	0.949	0.989	0.704	1.389
2020	−0.972	0.243	<0.001	0.378	0.235	0.610
2021	−0.767	0.189	<0.001	0.464	0.321	0.673
2022	−0.080	0.156	0.608	0.923	0.680	1.253
Monitoring sites						
Poultry farm	−1.990	0.269	<0.001	0.137	0.081	0.231
Poultry free-range sites	−2.089	0.232	<0.001	0.124	0.079	0.195
Mobile vendors	−0.471	0.202	0.020	0.625	0.421	0.927
Slaughterhouse	−1.158	0.279	<0.001	0.314	0.182	0.543
Sample types						
Chopping board	0.857	0.200	<0.001	2.357	1.592	3.488
Feces	−0.063	0.159	0.692	0.939	0.688	1.282
Sewage	0.132	0.187	0.482	1.141	0.790	1.647
Drinking water	−0.110	0.193	0.570	0.896	0.614	1.308
Others	−0.411	0.437	0.348	0.663	0.281	1.563
Birds origins						
Autotrophy	−1.058	0.170	<0.001	0.347	0.249	0.484

The first category was used as the reference group for all four independent variables. The year 2018 was selected as the reference group for “Year” group. The live poultry market was selected as the reference group for “Monitoring site” group. The AIV had not been detected in wild bird habitats and the result is not shown here. The cage was selected as the reference group for “Sample types” group. Trafficking sample was selected as the reference group for “Birds origins” group. The AIV had not been detected in wild origin samples and the result is not shown here. *β*, regression coefficient; SE, standard error; OR, odds ratio; and CI, confidence interval.

## Data Availability

All relevant data are within the manuscript and its additional files.

## Abbreviations

IAV: Influenza A virus
AIV: Avian influenza virus
COVID-19: Coronavirus disease-2019
NPIs: Nonpharmaceutical interventions
SD: Standard deviation
*β*: Regression coefficient
SE: Standard error
OR: Odds ratio
CI: Confidence interval.

## Supplementary Materials

Supplementary Materials AIV nucleic acid positive rate of different monitoring sites, sample types and samples from birds of different origins.
