# Supplementary material for: Effect of Nonpharmaceutical Interventions in Preventing COVID-19 on the Circulation of Avian Influenza Virus in Wuhan, Hubei Province, China
Source: Transbound Emerg Dis. 2024 May 25;2024:5528986. doi: 10.1155/2024/5528986 (PMC12019926; doi:10.1155/2024/5528986)
Supplement: Supplementary Materials — AIV nucleic acid positive rate of different monitoring sites, sample types and samples from birds of different origins. [file 5528986.f1.docx]

**Supplementary material**

**Table S1 H5, H9, Untyped and coexistence of H5 and H9 nucleic acid positive rate of different monitoring sites [positive rates (%) = (number of positive samples/number of samples collected) × 100].**

| Categories | Live poultry markets | Poultry farm | Poultry free-range sites | Mobile vendors | Slaughterhouse | Wild bird habitat |
| --- | --- | --- | --- | --- | --- | --- |
| AIV |  |  |  |  |  |  |
| 2018 | 45.49 (126/277) | 10.11 (9/89) | 12.57 (21/167) | 36.36 (28/77) | 10.00 (1/10) | 0 (0/41) |
| 2019 | 55.09 (92/167) | 0 (0/10) | 0 (0/37) | 17.39 (8/46) | 25.00 (5/20) | 0 (0/40) |
| 2020 | 31.03 (27/87) | 0 (0/32) | 0 (0/97) | 15.79 (3/19) | 0 (0/5) | 0 (0/30) |
| 2021 | 36.99 (54/146) | 13.51 (5/37) | 0.65 (1/153) | 8.33 (2/24) | 20.00 (2/10) | 0 (0/40) |
| 2022 | 47.20 (101/214) | 4.48 (3/67) | 3.60 (14/389) | 60.00 (12/20) | 43.33 (13/30) | 0 (0/60) |
| Total | 44.89 (400/891) | 7.23 (17/235) | 4.27 (36/843) | 28.49 (53/186) | 28.00 (21/75) | 0 (0/211) |
| Mean ± SD | 43.16 ± 9.34 | 5.62 ± 6.06 | 3.36 ± 5.36 | 27.57 ± 20.87 | 19.67 ± 16.35 | / |
| A/H5 |  |  |  |  |  |  |
| 2018 | 6.50(18/277) | 0(0/89) | 0(0/167) | 6.49(5/77) | 0(0/10) | 0(0/41) |
| 2019 | 8.38(14/167) | 0(0/10) | 0(0/37) | 2.17(1/46) | 0(0/20) | 0(0/40) |
| 2020 | 4.60(4/87) | 0(0/32) | 0(0/97) | 0(0/19) | 0(0/5) | 0(0/30) |
| 2021 | 0(0/146) | 0(0/37) | 0(0/153) | 0(0/24) | 0(0/10) | 0(0/40) |
| 2022 | 6.54(14/214) | 0(0/67) | 0(0/389) | 0(0/20) | 0(0/30) | 0(0/60) |
| Total | 5.61(50/891) | 0(0/235) | 0(0/843) | 3.23(6/186) | 0(0/75) | 0(0/211) |
| Mean ± SD | 5.20 ± 3.20 | / | / | 1.73 ± 2.82 | / | / |
| A/H9 |  |  |  |  |  |  |
| 2018 | 20.94(58/277) | 3.37(3/89) | 8.38(14/167) | 18.18(14/77) | 0(0/10) | 0(0/41) |
| 2019 | 31.74(53/167) | 0(0/10) | 0(0/37) | 6.52(3/46) | 25.00(5/20) | 0(0/40) |
| 2020 | 24.14(21/87) | 0(0/32) | 0(0/97) | 15.79(3/19) | 0(0/5) | 0(0/30) |
| 2021 | 35.62(52/146) | 13.51(5/37) | 0.65(1/153) | 8.33(2/24) | 0(0/10) | 0(0/40) |
| 2022 | 23.36(50/214) | 2.99(2/67) | 3.34(13/389) | 50.00(10/20) | 40.00(12/30) | 0(0/60) |
| Total | 26.26(234/891) | 4.26(10/235) | 3.32(28/843) | 17.20(32/186) | 22.67(17/75) | 0(0/211) |
| Mean ± SD | 27.16 ± 6.22 | 3.97 ± 5.56 | 2.47 ± 3.58 | 19.76 ± 17.60 | 13 ± 18.57 | / |
| A/Untyped |  |  |  |  |  |  |
| 2018 | 10.83(30/277) | 6.74(6/89) | 4.19(7/167) | 9.09(7/77) | 10.00(1/10) | 0(0/41) |
| 2019 | 3.59(6/167) | 0(0/10) | 0(0/37) | 2.17(1/46) | 0(0/20) | 0(0/40) |
| 2020 | 0(0/87) | 0(0/32) | 0(0/97) | 0(0/19) | 0(0/5) | 0(0/30) |
| 2021 | 0.68(1/146) | 0(0/37) | 0(0/153) | 0(0/24) | 20.00(2/10) | 0(0/40) |
| 2022 | 6.07(13/214) | 1.49(1/67) | 0.26(1/389) | 10.00(2/20) | 3.33(1/30) | 0(0/60) |
| Total | 5.61(50/891) | 2.98(7/235) | 0.95(8/843) | 5.38(10/186) | 5.33(4/75) | 0(0/211) |
| Mean ± SD | 4.23 ± 4.41 | 1.65 ± 2.92 | 0.89 ± 1.85 | 4.25 ± 4.92 | 6.67 ± 8.50 | / |
| A/H5 + H9 |  |  |  |  |  |  |
| 2018 | 7.22(20/277) | 0(0/89) | 0(0/167) | 2.60(2/77) | 0(0/10) | 0(0/41) |
| 2019 | 11.38(19/167) | 0(0/10) | 0(0/37) | 6.52(3/46) | 0(0/20) | 0(0/40) |
| 2020 | 2.30(2/87) | 0(0/32) | 0(0/97) | 0(0/19) | 0(0/5) | 0(0/30) |
| 2021 | 0.68(1/146) | 0(0/37) | 0(0/153) | 0(0/24) | 0(0/10) | 0(0/40) |
| 2022 | 11.21(24/214) | 0(0/67) | 0(0/389) | 0(0/20) | 0(0/30) | 0(0/60) |
| Total | 7.41(66/891) | 0(0/235) | 0(0/843) | 2.69(5/186) | 0(0/75) | 0(0/211) |
| Mean ± SD | 6.56 ± 4.95 | / | / | 1.82 ± 2.86 | / | / |

The non-H5/7/9 subtypes were referred to as Untyped.

SD, Standard deviation.

**Table. S2 H5, H9, Untyped and coexistence of H5 and H9 nucleic acid positive rate of different sample types [positive rates (%) = (number of positive samples/number of samples collected) × 100].**

| Categories | Cage | Chopping board | Feces | Sewage | Drinking water | Others |
| --- | --- | --- | --- | --- | --- | --- |
| AIV |  |  |  |  |  |  |
| 2018 | 25.51 (50/196) | 66.00 (33/50) | 21.76 (52/239) | 48.08 (25/52) | 23.39 (29/124) | 10.00 (1/10) |
| 2019 | 29.33 (22/75) | 42.86 (6/14) | 29.75 (36/121) | 43.33 (26/60) | 33.33 (15/45) | 0 (0/5) |
| 2020 | 20.69 (12/58) | 19.05 (4/21) | 9.88 (8/81) | 10.53 (4/38) | 2.86 (1/35) | 2.70 (1/37) |
| 2021 | 16.30 (15/92) | 54.05 (20/37) | 7.58 (10/132) | 12.50 (8/64) | 15.87 (10/63) | 4.55 (1/22) |
| 2022 | 15.03 (26/173) | 38.14 (37/97) | 16.59 (37/223) | 21.43 (24/112) | 8.55 (13/152) | 26.09 (6/23) |
| Total | 21.04 (125/594) | 45.66 (100/219) | 17.96 (143/796) | 26.69 (87/326) | 16.23 (68/419) | 9.28 (9/97) |
| Mean ± SD | 21.37 ± 6.06 | 44.02 ± 17.63 | 17.11 ± 9.01 | 27.17 ± 17.49 | 16.80 ± 12.04 | 8.67 ± 10.40 |
| A/H5 |  |  |  |  |  |  |
| 2018 | 2.04(4/196) | 6.00(3/50) | 3.77(9/239) | 7.69(4/52) | 2.42(3/124) | 0(0/10) |
| 2019 | 4.00(3/75) | 7.14(1/14) | 1.65(2/121) | 11.67(7/60) | 4.44(2/45) | 0(0/5) |
| 2020 | 3.45(2/58) | 0(0/21) | 1.23(1/81) | 0(0/38) | 2.86(1/35) | 0(0/37) |
| 2021 | 0(0/92) | 0(0/37) | 0(0/132) | 0(0/64) | 0(0/63) | 0(0/22) |
| 2022 | 1.16(2/173) | 3.09(3/97) | 0.90(2/223) | 1.79(2/112) | 3.29(5/152) | 0(0/23) |
| Total | 1.85(11/594) | 3.20(7/219) | 1.76(14/796) | 3.99(13/326) | 2.63(11/419) | 0(0/97) |
| Mean ± SD | 2.13 ± 1.64 | 3.25 ± 3.31 | 1.51 ± 1.40 | 4.23 ± 5.22 | 2.60 ± 1.64 | / |
| A/H9 |  |  |  |  |  |  |
| 2018 | 14.29(28/196) | 44.00(22/50) | 11.30(27/239) | 7.69(4/52) | 8.06(10/124) | 0(0/10) |
| 2019 | 16.00(12/75) | 28.57(4/14) | 20.66(25/121) | 13.33(8/60) | 26.67(12/45) | 0(0/5) |
| 2020 | 15.52(9/58) | 19.05(4/21) | 7.41(6/81) | 10.53(4/38) | 0(0/35) | 2.70(1/37) |
| 2021 | 14.13(13/92) | 51.35(19/37) | 7.58(10/132) | 10.94(7/64) | 15.87(10/63) | 4.55(1/22) |
| 2022 | 10.40(18/173) | 22.68(22/97) | 11.21(25/223) | 10.71(12/112) | 4.61(7/152) | 13.04(3/23) |
| Total | 13.47(80/594) | 32.42(71/219) | 11.68(93/796) | 10.74(35/326) | 9.31(39/419) | 5.15(5/97) |
| Mean ± SD | 14.07 ± 2.20 | 33.13 ± 13.95 | 11.63 ± 5.39 | 10.64 ± 2.00 | 11.04 ± 10.48 | 4.06 ± 5.38 |
| A/Untyped |  |  |  |  |  |  |
| 2018 | 6.63(13/196) | 12.00(6/50) | 5.02(12/239) | 9.62(5/52) | 12.10(15/124) | 0(0/10) |
| 2019 | 4.00(3/75) | 0(0/14) | 1.65(2/121) | 1.67(1/60) | 2.22(1/45) | 0(0/5) |
| 2020 | 0(0/58) | 0(0/21) | 0(0/81) | 0(0/38) | 0(0/35) | 0(0/37) |
| 2021 | 1.09(1/92) | 2.70(1/37) | 0(0/132) | 1.56(1/64) | 0(0/63) | 0(0/22) |
| 2022 | 0.58(1/173) | 7.22(7/97) | 2.69(6/223) | 2.68(3/112) | 0.66(1/152) | 0(0/23) |
| Total | 2.86(17/594) | 6.39(14/219) | 2.64(21/796) | 3.07(10/326) | 4.06(17/419) | 0(0/97) |
| Mean ± SD | 2.46 ± 2.79 | 4.38 ± 5.18 | 1.87 ± 2.10 | 3.11 ± 3.77 | 3.00 ± 5.17 | / |
| A/H5 + H9 |  |  |  |  |  |  |
| 2018 | 2.55(5/196) | 4.00(2/50) | 1.67(4/239) | 23.08(12/52) | 0.81(1/124) | 10.00(1/10) |
| 2019 | 5.33(4/75) | 7.14(1/14) | 5.79(7/121) | 16.67(10/60) | 0(0/45) | 0(0/5) |
| 2020 | 1.72(1/58) | 0(0/21) | 1.23(1/81) | 0(0/38) | 0(0/35) | 0(0/37) |
| 2021 | 1.09(1/92) | 0(0/37) | 0(0/132) | 0(0/64) | 0(0/63) | 0(0/22) |
| 2022 | 2.89(5/173) | 5.15(5/97) | 1.79(4/223) | 6.25(7/112) | 0(0/152) | 13.04(3/23) |
| Total | 2.69(16/594) | 3.65(8/219) | 2.01(16/796) | 8.90(29/326) | 0.24(1/419) | 4.12(4/97) |
| Mean ± SD | 2.72 ± 1.62 | 3.26 ± 3.18 | 2.10 ± 2.18 | 9.20 ± 10.33 | 0.16 ± 0.36 | 4.61 ± 6.40 |

The non-H5/7/9 subtypes were referred to as Untyped.

SD, Standard deviation.

**Table. S3 H5, H9, Untyped and coexistence of H5 and H9 nucleic acid positive rate of samples from birds of different origins [positive rates (%) = (number of positive samples/number of samples collected) × 100].**

| Categories | Trafficking | Autotrophy | Wild |
| --- | --- | --- | --- |
| AIV |  |  |  |
| 2018 | 40.58 (127/313) | 9.89 (26/263) | 0 (0/41) |
| 2019 | 51.87 (83/160) | 12.77 (12/94) | 0 (0/40) |
| 2020 | 27.27 (24/88) | 3.95 (6/152) | 0 (0/30) |
| 2021 | 27.74 (43/155) | 7.18 (14/195) | 0 (0/40) |
| 2022 | 44.32 (117/264) | 5.70 (26/456) | 0 (0/60) |
| Total | 40.20 (394/980) | 7.24 (84/1,160) | 0 (0/211) |
| Mean ± SD | 38.36 ± 10.71 | 7.90 ± 3.49 | / |
| A/H5 |  |  |  |
| 2018 | 6.71(21/313) | 0.76(2/263) | 0(0/41) |
| 2019 | 6.87(11/160) | 0(0/94) | 0(0/40) |
| 2020 | 4.55(4/88) | 0(0/152) | 0(0/30) |
| 2021 | 0(0/155) | 0(0/195) | 0(0/40) |
| 2022 | 5.30(14/264) | 0(0/456) | 0(0/60) |
| Total | 5.10(50/980) | 0.17(2/1,160) | 0(0/211) |
| Mean ± SD | 4.69 ± 2.79 | 0.15 ± 0.34 | / |
| A/H9 |  |  |  |
| 2018 | 16.93(53/313) | 5.70(15/263) | 0(0/41) |
| 2019 | 30.00(48/160) | 10.64(10/94) | 0(0/40) |
| 2020 | 20.45(18/88) | 3.95(6/152) | 0(0/30) |
| 2021 | 25.16(39/155) | 7.18(14/195) | 0(0/40) |
| 2022 | 24.62(65/264) | 4.82(22/456) | 0(0/60) |
| Total | 22.76(223/980) | 5.78(67/1,160) | 0(0/211) |
| Mean ± SD | 23.43 ± 4.97 | 6.46 ± 2.62 | / |
| A/Untyped |  |  |  |
| 2018 | 10.86(34/313) | 2.28(6/263) | 0(0/41) |
| 2019 | 3.12(5/160) | 2.13(2/94) | 0(0/40) |
| 2020 | 0(0/88) | 0(0/152) | 0(0/30) |
| 2021 | 1.94(3/155) | 0(0/195) | 0(0/40) |
| 2022 | 5.30(14/264) | 0.88(4/456) | 0(0/60) |
| Total | 5.71(56/980) | 1.03(12/1,160) | 0(0/211) |
| Mean ± SD | 4.24 ± 4.17 | 1.06 ± 1.11 | / |
| A/H5 + H9 |  |  |  |
| 2018 | 6.07(19/313) | 1.14(3/263) | 0(0/41) |
| 2019 | 11.87(19/160) | 0(0/94) | 0(0/40) |
| 2020 | 2.27(2/88) | 0(0/152) | 0(0/30) |
| 2021 | 0.65(1/155) | 0(0/195) | 0(0/40) |
| 2022 | 9.09(24/264) | 0(0/456) | 0(0/60) |
| Total | 6.63(65/980) | 0.26(3/1,160) | 0(0/211) |
| Mean ± SD | 5.99 ± 4.65 | 0.23 ± 0.51 | / |

The non-H5/7/9 subtypes were referred to as Untyped.

SD, Standard deviation.
